# A non-coding GWAS variant impacts anthracycline-induced cardiotoxic phenotypes in human iPSC-derived cardiomyocytes

**DOI:** 10.1038/s41467-022-34917-y

**Published:** 2022-11-22

**Authors:** Xi Wu, Fei Shen, Guanglong Jiang, Gloria Xue, Santosh Philips, Laura Gardner, Geneva Cunningham, Casey Bales, Erica Cantor, Bryan Paul Schneider

**Affiliations:** Department of Hematology and Oncology, Indiana University School of Medicine, Indianapolis, IN 46202 USA

**Keywords:** Cardiovascular genetics, Gene regulation, Mechanisms of disease, Transcriptomics

## Abstract

Anthracyclines, widely used to treat breast cancer, have the potential for cardiotoxicity. We have previously identified and validated a germline single nucleotide polymorphism, *rs28714259*, associated with an increased risk of anthracycline-induced heart failure. We now provide insights into the mechanism by which *rs28714259* might confer increased risk of cardiac damage. Using hiPSC-derived cardiomyocyte cell lines with either intrinsic polymorphism or CRISPR-Cas9-mediated deletion of *rs28714259* locus, we demonstrate that glucocorticoid receptor signaling activated by dexamethasone pretreatment prior to doxorubicin exposure preserves cardiomyocyte viability and contractility in cardiomyocytes containing the major allele. Homozygous loss of the *rs28714259* major allele diminishes dexamethasone’s protective effect. We further demonstrate that the risk allele of *rs28714259* disrupts glucocorticoid receptor and *rs28714259* binding affinity. Finally, we highlight the activation of genes and pathways involved in cardiac hypertrophy signaling that are blocked by the risk allele, suggesting a decreased adaptive survival response to doxorubicin-related stress.

## Abstract (web summary)

Germline variants may pre-dispose patients to an increased risk of developing anthracycline-induced cardiotoxicity. This report provides insights into the mechanism by which a common genetic variant, rs28714259, may confer an increased risk of cardiac damage.

## Introduction

Anthracyclines are commonly used to treat a diverse array of hematological and solid tumors^1^. Despite the development of novel targeted therapies, recent data demonstrated that anthracyclines remain a critical component for optimal outcomes in the curative setting for patients with biologically aggressive breast cancer^2^. These benefits, however, must be considered in context of toxicity, such as heart failure (HF), which is seen in about 2% of breast cancer patients^3^. The typical phenotype of HF is associated with a decreased left ventricular ejection fraction with dilated cardiomyopathy and is generally irreversible^4^. While cumulative dose and older age are established risk factors for development of anthracycline-induced HF, the risk attribution for many cases remains unclear^5,6^. Despite efforts to determine risk factors, develop less toxic derivatives, and identify subclinical toxicity earlier, there is no consensus on the best approach to prevent anthracycline-induced cardiotoxicity (AIC)^7–11^. The exact mechanism of this complication also has yet to be completely elucidated. Prior studies have suggested the contributing role of topoisomerase 2β inhibition, generation of oxidative stress, mitochondrial dysfunction, and defects in iron and calcium handling^7,12–17^. Prior studies have also suggested a potential for an inherited predisposition to AIC, although very few have demonstrated functionality of the candidate genetic variant associated with risk in a disease-relevant model^14,18–21^. Previously, our group identified the association of a non-coding single nucleotide polymorphism (SNP), *rs28714259* (G/A), with risk of AIC through a genome-wide association study (GWAS) in a 5000 patient, randomized phase III adjuvant breast cancer trial, E5103 (OR = 2.1; *p* = 9.25 × 10^−6^)^22^. The variant, *rs28714259*, was then validated in two independent phase III adjuvant breast cancer trials: E1199 and BEATRICE (OR = 1.9; *p* = 0.04, and OR = 4.2; *p* = 0.018, respectively). Recently, the association of rs287142559 with the risk of AIC was validated in an independent dataset (OR = 4.2; *p* = 0.006) by other investigators^23^. Herein, we explored the role of *rs28714259* polymorphism in AIC utilizing a clinically relevant model of hiPSC-derived cardiomyocytes (hiPSC-CMs). Leveraging hiPSC-CMs intrinsically polymorphic at *rs28714259* and by genome modification with CRISPR-Cas9, we demonstrate the impact of *rs28714259* on cardiomyocyte survival and function following doxorubicin exposure. We also provide mechanistic support for the role of *rs28714259* as part of a novel transcription enhancer site and the key signaling pathways impacted by the risk variant with RNA-seq analysis.

## Results

### rs28714259 risk allele (A) disrupts glucocorticoid receptor binding in vitro and in vivo

**Fig. 1:**
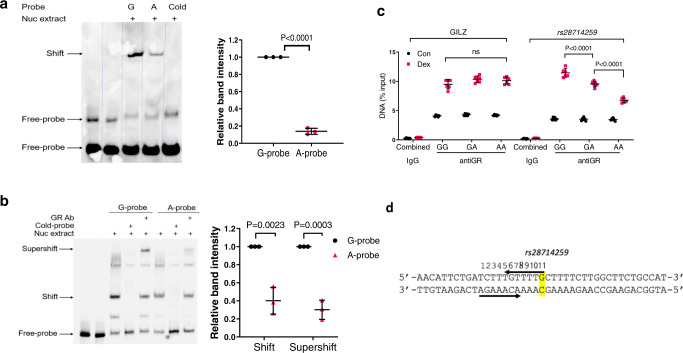
*rs28714259* risk allele or deletion decreases glucocorticoid receptor (GR)-mediated enhancer activity and disrupts GR binding. **a** GR:*rs28714259* binding efficiency determined by Electrophoretic Mobility Shift Assay (EMSA) in hiPSC-derived cardiomyocyte cells (hiPSC-CMs). Left: Representative gel image. Right: quantification of band intensities. **b** GR:*rs28714259* binding efficiency determined by EMSA in MCF-7 breast cancer cells. Left: Representative gel image. Right: quantification of band intensities. To induce GR binding, cells were treated with 100 nM dexamethasone for 2 h and nuclear extract was incubated with biotin-labeled, G (Major)- or A (Risk)-probes. GR antibody was added to the binding reaction to detect the super shift band. **a**, **b** Three independent experiments were performed in hiPSC-CMs or MCF-7 cells for quantification (*n* = 3). **c** GR:*rs28714259* binding efficiency in hiPSC-CMs of each genotype (intrinsic lines) determined by Chromatin Immunoprecipitation (ChIP) assay. **d** Schematic of *rs28714259* (position 11, yellow highlight) location. GILZ, a known GR-mediated transcriptional enhancer region, was used as positive control. For rabbit IgG control, data from hiPSC-CMs of GG, GA and AA genotypes were combined (*n* = 6). Two pull-downs were performed using three independent lines for each genotype (GG/GA/AA) (*n* = 6). Data are mean ± sd. Two-tailed unpaired *t*-tests (**a**–**c**), or two-way ANOVA followed by Tukey’s multiple comparisons test (**d**), were used to estimate significance. *P*-values are indicated in the figure. Source data for Fig. 1a–c are provided as a Source Data file.

Our in silico analysis suggests that *rs28714259* locates in a potential glucocorticoid receptor (GR) binding site, consistent with ChIP-Seq data from the ENCODE project suggesting *rs28714259*’s location within a 300 bp sequence pulled down by GR antibody^24,25^ and moreover, predicts that the risk (A) allele disrupts potential GR binding. To verify allele A disrupts (decreases) binding efficiency of GR to the *rs28714259* locus containing the A-variant, we performed an electrophoretic mobility shift assay (EMSA) using nuclear extract from a hiPSC-CM line of GG genotype treated with dexamethasone to induce GR nuclear translocation. The *rs28714259* G allele-probe binds strongly with cardiomyocyte nuclear protein stimulated by dexamethasone, as evidenced by the prominent band shift (Fig. 1a). Notably, we observe an 80% reduction in the band intensity of risk allele (A)-probes compared to major allele (G)-probes, suggesting decreased GR binding to risk allele probes. We also show in vitro binding of GR to *rs28714259* probes using nuclear extract from dexamethasone-treated MCF-7 breast cancer cells as these cells have high levels of GR expression (Supplementary Fig. 1). Consistently, we observe reduced band intensity in risk allele (A)-probes compared to major allele (G)-probes using dexamethasone-stimulated MCF-7 nuclear protein. Adding GR antibody to the binding reaction results in a detectable “supershift” which confirms that the nuclear protein binding to G and A allele-probe is indeed GR (Fig. 1b). Consistently, the supershift band intensity of the A allele-probe is decreased by over 60% compared to the G allele-probe. These results suggest that the risk (A) allele binds GR with less affinity than the major allele (G).

To corroborate our EMSA result, we performed Chromatin Immunoprecipitation (ChIP) assay to examine the impact of the risk (A)-allele on GR:*rs28714259* locus binding efficiency in vivo. Three hiPSC-CM lines intrinsically polymorphic at *rs28714259* (one for each genotype) were pretreated with dexamethasone and DNA was pulled down by a GR antibody. In cardiomyocytes homozygous of the major (G)-allele, dexamethasone stimulated GR binding to the *rs28714259* locus in a similar fashion to a known GR-binding site in the promoter region of the glucocorticoid induced leucine zipper (GILZ) gene (Fig. 1c)^26^. In comparison, GR:*rs28714259* binding is significantly decreased in heterozygous cardiomyocytes (GA) and further decreased but not completely abolished, in risk (A)-allele homozygous cells (AA). These results are consistent with the reduced binding to A allele-probes demonstrated by EMSA. Taken together, these data indicate that the presence of the risk (A)-allele indeed disrupts GR:*rs28714259* binding both in vitro and in vivo.

Comparing the known specific sequence of glucocorticoid response elements (GREs), the sequence around *rs28714259* (Fig. 1d) does not have the 3-bp spacer in GREs^27^. In addition, it has an extra nucleotide in each potential GR binding half sites and the two half sites are located on opposite strands. The similarity and dissimilarity between *rs28714259* sequence and GREs are shown by Supplementary Fig. 2.

### Loss of rs28714259 major allele or locus decreases GR-induced protection of viability and contractility in hiPSC-CMs following doxorubicin exposure

**Fig. 2:**
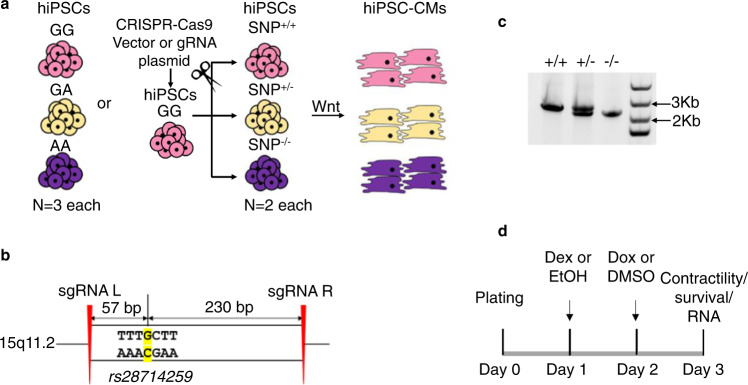
Generation of hiPSC-derived cardiomyocytes (iPSC-CMs) from *rs28714259* intrinsically polymorphic or deleted hiPSCs. **a** Schematic of hiPSC-CMs generation from *rs28714259* intrinsically polymorphic or CRISPR-Cas9-mediated deleted hiPSCs. **b** Schematic of sgRNA design for the deletion of the *rs28714259* region in hiPSCs. **c** Deletion of the variant region generates an amplicon 300 bp shorter than the amplicon from un-edited DNA.+/+, vector transfected clone with intact *rs28714259* region. +/−, one clone of heterozygous *rs28714259* region deletion. −/−, one clone of homozygous *rs28714259* region deletion. At least three independent experiments were repeated with similar results. **d** Treatment and assay timeline of hiPSC-CMs. Source data for Fig. 2c are provided as a Source Data file.

**Fig. 3:**
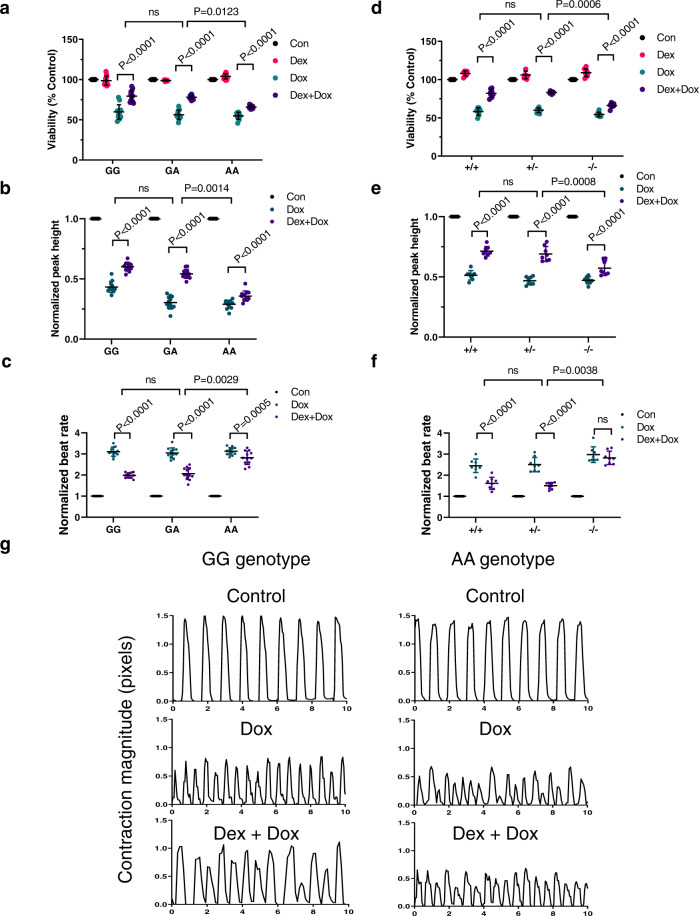
*rs28714259* risk allele or loss of *rs28714259* locus decreases dexamethasone -induced protection of viability and contractility after doxorubicin exposure. Cells were pretreated with or without dexamethasone before exposed to doxorubicin. Viability (**a**), normalized peak height (contractile strength) (**b**), and normalized beat rate (**c**) were evaluated in intrinsically polymorphic hiPSC-derived cardiomyocyte cell (hiPSC-CM) lines. Viability (**d**), normalized peak height (**e**), and normalized beat rate (**f**) were also assessed in *rs28714259* deleted hiPSC-CM lines. **g** Representative curves of contraction amplitude in two *rs28714259* intrinsically polymorphic hiPSC-CM lines of GG (left) and AA (right) genotypes, respectively. Contractility was analyzed using Cellogy Pulse from recorded movies of beating cardiomyocytes. *rs28714259* intrinsically polymorphic lines are labeled as GG, GA, and AA; *rs28714259* deletion lines are labeled as +/+, +/−, and −/−. Three lines/genotype for the intrinsically polymorphic hiPSC-CMs (GG/GA/AA) and two lines/genotype for genome-modified hiPSC-CMs [(+/+)/(+/−)/(−/−)] were measured with four technical replicates for each line. Results for each treatment in the intrinsic (*n* = 12) and genome-modified (*n* = 8) lines are plotted (dots). Data are represented as mean ± sd. Two-way ANOVA followed by Tukey’s multiple comparisons test was used to estimate significance. *P*-values are indicated in the figure. Con control, DOX doxorubicin, DEX dexamethasone. Source data for Fig. 3a–f are provided as a Source Data file.

Since our EMSA and ChIP assay results indicate that the *rs28714259* risk (A) allele disrupts the regulation of GR-induced protective signaling otherwise mediated by the major (G) allele against anthracycline-induced cardiotoxicity, we examined the impact of the AIC-associated *rs28714259* polymorphism on cardiomyocyte viability in vitro using human induced pluripotent stem cell-derived cardiomyocytes (hiPSC-CMs) induced from hiPSC cell lines intrinsically polymorphic at *rs28714259* and cell lines with *rs28714259*-deletion via CRISPR-Cas9 (Fig. 2a–c and Supplementary Fig. 3). Three intrinsically polymorphic hiPSC-CM lines per genotype (GG/GA/AA, 9 lines total) were pretreated with ethanol vehicle or 100 nM dexamethasone for 24 h before doxorubicin exposure, as the pretreatment with this concentration is known to activate GR signaling in various cell types^28,29^ and more importantly, crucial for protection against doxorubicin-induced cardiotoxicity^30^. Following dexamethasone or vehicle pre-treatment, hiPSC-CMs were treated with DMSO or 1 µM doxorubicin for 24 h, a dose compatible with clinical plasma doxorubicin concentration^31,32^ (Fig. 2d). Doxorubicin treatment reduces viability of hiPSC-CMs to ~60%, regardless of genotype. However, dexamethasone pre-treatment retains at least 80% viability in hiPSC-CMs homozygous and heterozygous of the major allele (GG and GA, respectively) (Fig. 3a), indicating activation of GR by dexamethasone resulting in a significant protective effect against doxorubicin-induced cell death. Of note, dexamethasone-induced protection against doxorubicin-induced cell death is significantly reduced in hiPSC-CMs homozygous of the risk allele (AA) (Fig. 3a).

As decreased left ventricular ejection fraction (LVEF) is a defining clinical manifestation of HF and glucocorticoid stimulation increases cardiac contractility in vitro^33^, we examined the effect of *rs28714259* polymorphism on GR-mediated cardiomyocyte contractility with live movies of beating hiPSC-CM syncytium (Supplementary Movies 1–6). Three intrinsically polymorphic hiPSC-CM lines per genotype (GG/GA/AA, 9 lines total) were pretreated with ethanol vehicle or dexamethasone, followed by DMSO vehicle or doxorubicin as described earlier. Compared to vehicle only exposure, doxorubicin treatment decreases contraction amplitude with the expected compensatory increase in beat rate for all genotypes (Fig. 3b, c). Dexamethasone pretreatment results in partial but significant protection from reduced doxorubicin-induced contractility and increased beat rate in *rs28714259* GG and GA cardiomyocytes. However, this protective effect is negligible in the cardiomyocytes homozygous with the *rs28714259* risk allele (AA) (Fig. 3b, c; Supplementary Movies 1–6).

To confirm the impact of the risk allele seen in the intrinsically polymorphic cell lines, we performed viability and contractility assays in *rs28714259* deleted hiPSC-CMs (Fig. 3d–f). Similarly, the protective effect of GR is significantly reduced in *rs28714259* homozygous deleted hiPSC-CMs (−/−) compared to vector control (+/+) or heterozygous deleted (+/−) cells, suggesting that the *rs28714259* locus is crucial for GR-induced viability retention with doxorubicin treatment. Dexamethasone pretreatment consistently rescues contraction amplitude and beat rate in the vector control and the heterozygous deleted cardiomyocytes, but has only minimal rescue effect in the homozygous deleted cells (Fig. 3e, f). These data indicate that *rs28714259* locus is a mediator of dexamethasone-induced conservation of cardiac contractility following doxorubicin exposure.

### rs28714259 risk allele (A) decreases GR-mediated enhancer activity

**Fig. 4:**
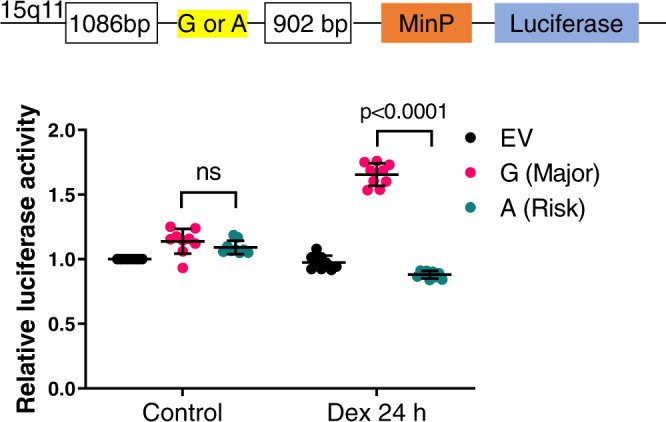
Luciferase activity in hiPSC-derived cardiomyocyte cells (hiPSC-CMs) transfected with empty vector (EV), major allele (G)-, or risk allele (A)-constructs. Schematic of the luciferase construct to test the enhancer activity of *rs28714259* locus (Top). Luciferase activity in transfected cells treated with vehicle or dexamethasone (bottom). Three independent experiments were performed with three technical repeats for each construct transfection (*n* = 9). Data are mean ± sd. Two-tailed unpaired *t*-tests were used to estimate significance. *P*-values are indicated in the figure. Source data are provided as a Source Data file.

Next, we tested whether *rs28714259* mediates dexamethasone-induced protection of cardiomyocyte viability and contractility through GR-mediated transcriptional regulation. We cloned a 2 kb DNA sequence centering around *rs28714259* (1 kb on both sides of the SNP) with either the major (G), or risk (A) allele into a luciferase reporter plasmid with a minimal promoter (Fig. 4) and transfected the constructs into major allele homozygote hiPSC-CMs. The cells were then treated with dexamethasone or ethanol vehicle control. Luciferase activity in hiPSC-CMs transfected with major (G) allele-construct is increased 1.5-fold after dexamethasone treatment compared to vehicle treated group, indicating that the *rs28714259* locus possesses cis-acting enhancer activity for GR-mediated transcriptional activation. In contrast, we did not observe a significant increase in luciferase activity by transfection of the risk (A)-allele construct following dexamethasone treatment, suggesting the risk allele disrupts the enhancer activity (Fig. 4).

#### rs28714259 risk allele impacts gene expressions in cardiac hypertrophy signaling following dexamethasone/doxorubicin combination treatment

**Fig. 5:**
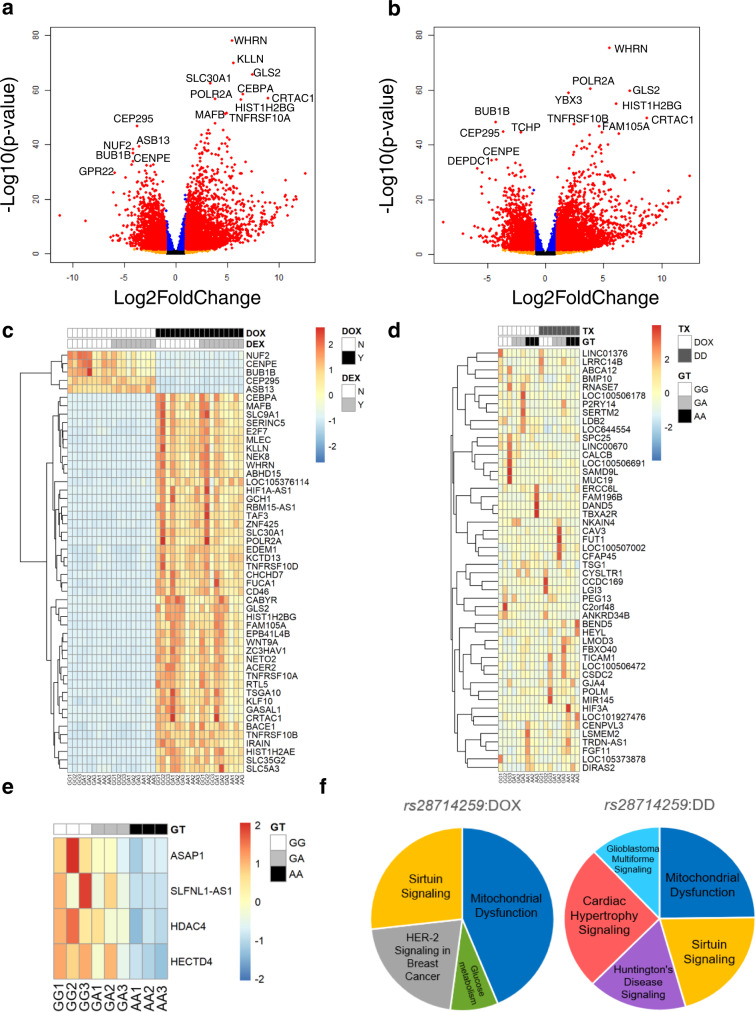
Transcriptomic changes induced by doxorubicin and dexamethasone/doxorubicin combination treatment and genes/pathways impacted by the *rs28714259* risk (A) allele. **a**, **b** Volcano plots representation of differential gene expression pattern induced by doxorubicin-only (**a**) or dexamethasone/doxorubicin combination treatment (**b**) over vehicle-treated control in nine *rs28714259* intrinsically polymorphic hiPSC-derived cardiomyocyte cell (hiPSC-CM) lines. Tests for differential expression were performed with DESeq2 package in R using a negative binomial generalized linear model (two-sided test). The *p*-values were then corrected for multiple hypothesis testing using the False Discovery rate (FDR) approach of Benjamini–Hochberg. An FDR < 0.05 were considered statistically significant. Significantly differentially expressed genes (FDR < 0.05) of Log2 Fold Change (|log2FC|) > 1 are highlighted in red, while those of |log2FC| < 1 are highlighted in blue. Black and orange highlights represent genes with FDR > 0.05. Top differentially expressed genes are labeled. **c** Heat map of gene expression patterns in vehicle control, dexamethasone-only, doxorubicin-only, and dexamethasone/doxorubicin combination treatment groups. Top 50 genes most differentially regulated by doxorubicin treatment (|Log2FC > 1|, ranked by *P*-value) are shown. **d** Heat map of top 50 most differentially regulated genes between doxorubicin-only and dexamethasone/doxorubicin combination treatments based on ΔLog2FC in nine *rs28714259* intrinsically polymorphic hiPSC-CM lines. **e** Heat map of differential gene expression induced by dexamethasone/doxorubicin combination treatment significantly impacted by the risk (A) allele. Differential gene expression after treatment for each sample was normalized to its baseline (control) expression (**d**, **e**). **f** Top pathways differentially regulated by the risk (A) allele following doxorubicin or dexamethasone and doxorubicin combination treatment identified by IPA. Benjamini-Hochberg (B-H) adjusted *p*-values were calculated in IPA to account for multiple comparisons, and the B-H adjusted *p*-value < 0.05 was considered as statistically significant. Percentage of pie chart is proportional to the number of genes identified in each pathway. Pathways are ordered clockwise by *P*-value. TX treatment, GT genotype, DOX doxorubicin, DD dexamethasone/doxorubicin combination. Source data for Fig. 5a–f are provided as a Source Data file.

Our findings using hiPSC-CM suggest that the *rs28714259* variant decrease the protective effect of dexamethasone pretreatment from doxorubicin-induced cell death and a decline in contractility. Since the *rs28714259* locus is capable of GR binding and activation of gene expression, we performed RNA-seq analysis in hiPSC-CM lines intrinsically polymorphic at *rs28714259* to identify genes regulated by their binding. Nine hiPSC-CM lines intrinsically polymorphic at *rs28714259* (3 lines for each genotype) were pretreated with ethanol vehicle or dexamethasone, followed by DMSO vehicle or doxorubicin treatment. We first examined the effect of doxorubicin with or without dexamethasone on the global transcriptome regardless of *rs28714259* genotype. Combining results from all nine hiPSC-CM lines, dexamethasone pretreatment alone significantly upregulates the expression of *FKBP5* (FK506 binding protein 5) and *ZBTB16* (Zinc finger and BTB domain containing 16), and both have previously been reported as dexamethasone target genes in cardiomyocytes^34–37^. Since dexamethasone treatment alone may affect gene expression independent of doxorubicin-induced damage, we focused on the comparison between doxorubicin-only and dexamethasone/doxorubicin combination treatment. Doxorubicin induces a pronounced global transcriptomic change in hiPSC-CMs over vehicle-treated baseline, with 2985 upregulated and 2370 downregulated genes (log2FC > 1, FDR < 0.05) (Fig. 5a). Consistent with a role in inducing apoptosis in cardiomyocytes following doxorubicin exposure, death receptor family genes FAS (Fas Cell Surface Death Receptor), TNFRSF10A (Tumor Necrosis Factor Receptor Superfamily Member 10a), 10B, 10C, and 10D are significantly upregulated (Supplementary Table 1A). Ingenuity Pathway Analysis (IPA) identified “p53 signaling and Role of BRCA1 in DNA damage response” as the top differentially regulated pathways following doxorubicin exposure compared to vehicle-treated baseline, consistent with other reports of doxorubicin-induced cardiotoxicity (Supplementary Table 1B)^38,39^. The global transcriptomic response to dexamethasone/doxorubicin combination over vehicle-treated baseline are similar to that induced by doxorubicin-only group and contains evident overlap (Fig. 5a–c). When comparing a gene’s response to dexamethasone/doxorubicin combination to doxorubicin-only treatment in the same intrinsically polymorphic hiPSC-CM cell line, we notice a pronounced difference in expression levels in genes both up- and down-regulated by doxorubicin (Fig. 5d). Since dexamethasone appears to protect from doxorubicin-induced cardiotoxicity, we hypothesize that these differences in gene induction levels between dexamethasone/doxorubicin combination and doxorubicin-only treatment contribute to, at least partly, the protective mechanisms of dexamethasone pretreatment. *GJA4* (Gap Junction Protein Alpha 4), *DAND5* (DAN Domain BMP Antagonist Family Member 5), and *BEND5* (BEN Domain Containing 5) are among the genes with the greatest difference in expression levels between the dexamethasone/doxorubicin combination and doxorubicin-only treatment groups (Supplementary Table 2A). In addition, we find cAMP- and G-Protein Coupled Receptor (GPCR)-mediated signaling among the top pathways differentially regulated between the dexamethasone/doxorubicin combination and the doxorubicin-only treatment (Supplementary Table 2B). Although not statistically significant after corrections (*p*-values 0.057 and 0.071), since both pathways are highly ranked and have a mutual role in mediating cardiomyocyte survival^40^, we further examined genes identified in these two pathways. The identified genes appear to lead to an enhanced GPCR/cAMP pathway activity following dexamethasone/doxorubicin combination compared to doxorubicin-only treatment (Supplementary Table 3), indicating a role in promoting cardiomyocyte survival through dexamethasone pretreatment.

**Fig. 6:**
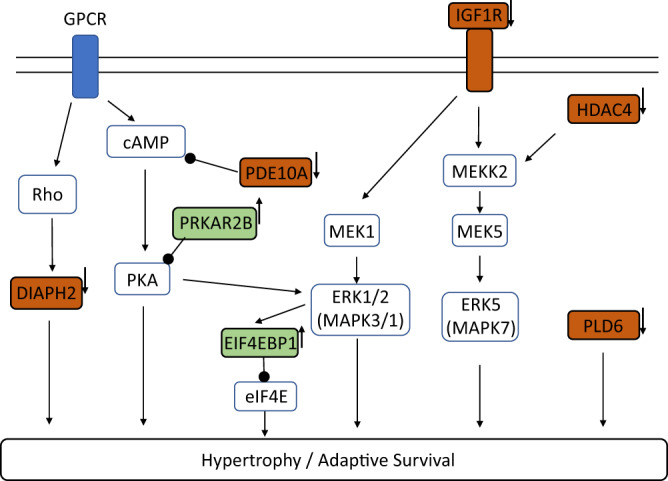
*rs28714259* risk (A) allele dysregulates genes following dexamethasone/doxorubicin combination treatment and contributes to an attenuated hypertrophy signaling. Top genes identified by IPA (in red or green blocks with bold black frame) are depicted in the context of canonical pathways. Red: pro-hypertrophic genes AND upregulated under dexamethasone/doxorubicin combination by *rs28714259* major (G) allele. Green: anti-hypertrophic genes AND down-regulated under dexamethasone/doxorubicin combination by *rs28714259* major (G) allele. Small arrows placed on the right side of identified genes represent the effect of *rs28714259* risk (A) allele, which consistently opposes the effect of major (G) allele. Only top genes (*P*-value <= 2.81 × 10−^3^) in the cardiac hypertrophy signaling pathway identified by Ingenuity Pathway Analysis are shown for simplicity. A complete list of the 31 genes identified is included in Supplementary Table 6.

Next, we examined the impact of *rs28714259* genotype on dexamethasone/doxorubicin-induced gene response using a two-way interaction analysis. *FKBP5* and *ZBTB16*, the top genes upregulated by dexamethasone-pretreatment alone, are not differentially regulated by the *rs28714259* genotype. Similarly, no single gene’s expression is found to be significantly impacted by *rs28714259* with doxorubicin treatment alone. However, at the network level, the risk (A)-allele demonstrates a significant impact on the ranking of critical pathways for cardiomyocyte function and survival following doxorubicin exposure, including mitochondrial dysfunction and glucose metabolism. (Supplementary Table 4). Since the protective effect of dexamethasone pretreatment is significantly attenuated by the risk (A) allele, we next evaluated the gene response to dexamethasone/doxorubicin combination treatment impacted by *rs28714259*. Four genes are significantly impacted by *rs28714259* by the combination treatment, including *ASAP1* (Arf-GAP with the SH3 domain, ANK repeat, and PH domain-containing protein 1), *SLFNL1-AS1* (Schlafen Like 1 Antisense RNA 1), *HDAC4* (Histone Deacetylases 4), and *HECTD4* (HECT Domain E3 Ubiquitin Protein Ligase 4). All four genes are up-regulated by the dexamethasone/doxorubicin combination treatment, but this induction is attenuated with the risk (A) allele (Fig. 5e and Supplementary Table 5). As expected, there is overlap between pathways dysregulated by *rs28714259* in the dexamethasone/doxorubicin combination group and those identified in the doxorubicin-only group (Fig. 5f and Supplementary Table 4). One pathway only dysregulated by the risk (A) allele, however, is cardiac hypertrophy signaling, indicative of its role in dexamethasone-pretreatment induced protective effect. 31 genes are highlighted in this pathway, including pro-hypertrophic genes such as *IGF1R* (Insulin Like Growth Factor 1 Receptor) and *HDAC4*, as well as anti-hypertrophic genes such as *PRKAR2B* (Protein Kinase CAMP-Dependent Type II Regulatory Subunit Beta) and *EIF4EBP1* (Eukaryotic Translation Initiation Factor 4E Binding Protein 1), some of which have potential interplay with known regulators of cardiomyocyte physiology (Fig. 6 and Supplementary Table 6). These genes appear to contribute to enhanced cardiac hypertrophy signaling induced by dexamethasone/doxorubicin combination treatment, but the enhancement is significantly attenuated by the risk (A) allele (Supplementary Table 6). These findings suggest that the risk (A) allele sub-optimally regulates a series of genes that appear to contribute to attenuated cardiac hypertrophy signaling.

## Discussion

Prior research has contributed to our understanding of AIC and a potential genetic contribution. Very few of the identified genetic variants, however, have been functionally validated using a disease relevant model. Our group identified and validated the association of a common germline SNP, *rs28714259* (G/A), with risk of anthracycline-induced HF through a GWAS in a large phase III adjuvant breast cancer trial with validation in two independent, phase III trials; findings also replicated by other investigators^22,23^. Here, using hiPSC-CM models with either intrinsic *rs28714259* polymorphisms or with deletion of the *rs28714259* locus, we provide evidence of *rs28714259*’s functional impact on cardiomyocyte survival and contractility. We further provide both in vitro and in vivo evidence that *rs28714259* may be a potential GR binding site capable of enhancing GR-mediated transcriptional regulation. In addition, we highlight key genes and signaling pathways impacted by the *rs28714259* risk variant. Together, our data provide insight into the mechanism by which the GWAS-identified AIC risk variant may lead to the clinical development of HF in patients. These data have the potential to better risk-stratify patients appropriate for doxorubicin-based therapy.

Dexamethasone is routinely used as a premedication for doxorubicin to help prevent an allergic infusion reaction and to help mitigate nausea and vomiting^41–43^. Serendipitously, prior pre-clinical data have shown that GR-signaling activated by dexamethasone pre-treatment may also play a role in minimizing potential cardiotoxicity^44,45^. Our results suggest that *rs28714259* is located in a potential GR binding site and the risk variant disrupts GR binding, indicating a possible intersection of *rs28714259* with GR binding and the risk of AIC. We further demonstrate that the *rs28714259* risk allele or loss of *rs28714259* locus does not affect cardiomyocyte survival and contractility with doxorubicin treatment alone, but significantly decreases the protective effect of dexamethasone pretreatment on cardiomyocyte survival and contractility (seen in both the intrinsic *rs28714259* polymorphic and the deleted hiPSC-CM models). Our in vitro findings which show decreased protective benefit of dexamethasone pretreatment for the *rs28714259* risk variant, further implicates the role of the variant in the development of anthracycline-induced HF through mediation of GR binding. In addition, our findings highlight the potential importance of steroid pretreatment on the prevention of cardiotoxicity, which could have implications for the growing group of anticancer drugs with cardiotoxicity. The known risk of heart failure for MEK inhibition^46–48^ also supports the finding of convergence of gene expression around the MEK pathway in this work. When considering the clinical implementation of these findings, one must not only consider the potential impact on the prevention of drug-toxicity, but also the potential impact on the tumor. Relevant to these findings, caution should be employed regarding the dose, duration, and type of glucocorticoid, as glucocorticoids have also been shown to promote breast cancer metastasis in mouse models^49^.

In agreement with an attenuated dexamethasone-induced protective effect conferred by the *rs28714259* risk allele, when looking at the genotype and drug interaction, we find the upregulation of four genes *ASAP1, SLFNL1-AS1, HDAC4, and HECTD4* by the dexamethasone pretreatment and doxorubicin combination to be significantly reduced by the risk allele. ASAP1 is best known for regulating focal adhesion by binding with actin filaments^50^. Other work has shown that knocking down *ASAP1* increases the activation of nuclear factor (NF)-κB following LPS-induced inflammatory response in macrophage-like cells^51^, suggesting a role of ASAP1 in repressing NF-κB. Anthracycline exposure in cardiomyocyte is known to induce an inflammatory response and NF-κB activation is pro-apoptotic^52^. Thus, reduced *ASAP1* expression by *rs28714259* following dexamethasone/doxorubicin combination may play a role in increased sensitivity to anthracyclines. HDAC4 has been shown to be pro-hypertrophic in myocardium of transgenic mice^53^. Dexamethasone treatment leads to cardiomyocyte hypertrophy in vitro, but also protected cells from TNF (tumor necrosis factor) -alpha induced apoptosis^54^, suggesting overlap between cardiac hypertrophy and survival signaling. It is plausible that *HDAC4* upregulation by dexamethasone/doxorubicin combination mediates part of a hypertrophic signaling as an adaptive survival response to doxorubicin-induced cell death. Congruent with these findings, using pathway analysis, we show 31 genes differentially regulated by the *rs28714259* risk variant with the dexamethasone/doxorubicin combination, including under-activation of pro-hypertrophic genes such as *HDAC4* and *IGF1R* (Insulin Like Growth Factor 1 Receptor), and reduced-inhibition of anti-hypertrophic genes such as *EIF4EBP1* (Eukaryotic Translation Initiation Factor 4E Binding Protein 1) and *PRKAR2B* (Protein Kinase CAMP-Dependent Type II Regulatory Subunit Beta). Together, these 31 genes differentially regulated by *rs28714259* appear to attenuate cardiac hypertrophy signaling with exposure to the dexamethasone/doxorubicin combination. Interestingly, many of these genes converge on known pro-hypertrophic pathways such as the GPCR/cAMP/PKA and MAPK/ERK1/2/ERK5 pathways^40,55–59^. Thus, we propose that *rs28714259* risk allele contributes to AIC through attenuation of important genes and pathways which would otherwise result in dexamethasone-induced cardiac hypertrophy-like signaling; potentially valuable for cardiomyocyte adaptation and survival. Future planned studies using circular chromosome conformation capture-Seq (4C-Seq) could further validate the four top target genes as well as provide insight into additional genes impacted by GR:*rs28714259* binding.

These findings merit further exploration of the role and possible clinical utility of genes significantly dysregulated by *rs28714259* such as *ASAP1* and *HDAC4*. Specifically, selective activation of *ASAP1* and *HDAC4* gene expression may be beneficial in the prevention and mitigation of AIC. Recently, the use of gene therapy to restore key protein levels via viral vectors and mRNA technology used for the treatment of heart failure have shown early promising results in both pre-clinical and clinical studies^60–62^. In addition, our pathway analysis suggests that AIC is likely not the result of a single aberrant gene but rather an intricate multigenic pathophysiology. Specifically, *rs28714259* dysregulates multiple genes important to attenuated cardiac hypertrophy signaling and their convergence on canonical pathways such as GPCR/cAMP/PKA and MAPK/ERK1/2/ERK5. Thus, strategic and selective activation of these pathways could provide another novel therapeutic approach to treat or prevent AIC. Indeed, administration of substrates that activates PKA or ERK1/2^63–65^ as well as activation of ERK5 by cardiac specific, constitutively-active MEK5^66^ have been shown to prevent doxorubicin-induced cardiotoxicity in pre-clinical models. Recent ERK5 inhibitors identified to paradoxically activate ERK5’s transcriptional activity provide a route for its pharmacological activation^67^. However, timing and duration of pathway activation are critical, as prolonged activation of canonical hypertrophy pathways may lead to cardiac remodeling and can worsen heart failure^40,55–59^. Another unique dilemma globally applicable to drug induced toxicity for cancer, is the competing concern of activating cancer survival pathways. Thus, strategies designed to target candidate pathways, must be developed to both abrogate the pathophysiological toxicity of the drug while not hindering its intended damage to the cancer cells. This intersection will likely require carefully executed studies with a focus on cardiac-specific delivery as well as the timing and duration of activation.

The current work employed *rs28714259* intrinsically polymorphic hiPSC-CMs to demonstrate the impact of the variant and were corroborated using cells lines with CRISPR-mediated variant region deletion. Although there are no other SNPs in tight linkage disequilibrium with *rs28714259* or any other known regulatory elements in the deleted region, it is possible that the deletion in our experiments may impact GR binding in unknown ways, such as the removal of sequences that facilitate GR cofactors binding. Future studies manipulating the *rs28714259* variant to the major allele in hiPSC-CM cell lines derived from patients who have actually experienced anthracycline-induced HF will help better understand the impact of *rs28714259* on cardiotoxicity and may provide other candidate genes regulated by *rs28714259*.

In conclusion, our results demonstrate that the GWAS-identified SNP, *rs28714259*, is linked to AIC through disruption of GR:*rs28714259* locus binding and its subsequent protective signaling. These findings, provide functional support for the consideration of *rs28714259* as a predictive biomarker for AIC. Further, this work highlights some key candidate genes and pathways for future studies focused on therapeutic mitigation of HF from anthracyclines. Glucocorticoids remain a standard therapy in the concurrent treatment with anthracyclines to prevent nausea and allergic reactions. Our findings highlight additional potential fortuitous benefits including the abrogation of anthracycline-induced cardiotoxicity. Future strategies to further capitalize on this pathway to further reduce the risk of heart failure is warranted. We recognize the complex interplay between preventing toxicity and activating cancer pathways^49^, and approaches must be balanced with great caution.

## Methods

The current study was approved by the Indiana University Institutional Review Board #1505859025.

### In silico analysis of *rs28714259* locus for transcription factor binding

In silico analysis was performed to examine the sequences surrounding *rs28714259* (G/A), a non-coding SNP, for any potential transcription factor binding site. The 40 bp sequences centered on *rs28714259* obtained from Genome Browser GRCh37/hg19 were analyzed with PROMO 3.0.2 (http://alggen.lsi.upc.es/cgi-bin/promo_v3/promo/promoinit.cgi?dirDB=TF_8.3)^68,69^. The sequences carrying either major (G) or risk (A) allele were loaded separately as the query sequence to search for potential binding sites. The prediction was carried out considering only human transcription factors.

### Human induced pluripotent stem cell lines and culture

hiPSC lines intrinsically polymorphic at *rs28714259* were generated by the iPSCORE resource^70^ and purchased from WiCell. *rs28714259* genotype information were determined based on the deposited whole genome sequencing results from National Center for Biotechnology Information dbGaP (the Database of Genotypes and Phenotypes, phs000924) with authorized access. Three cell lines, each of *rs28714259* GG and GA genotypes, and two cell lines of the AA genotype from healthy female donors were acquired and renamed GG 1-3, GA 1-3, and AA 2-3, respectively. The original names of the cell lines and their *rs28714259* genotype may be shared with other researchers with independent, approved Project Request from their institutions for access to phs000924. *rs28714259* genotype of individual cell lines was confirmed using TaqMan SNP Genotyping Assays (#4351379, Thermo Fisher Scientific). Due to the limited availability of hiPSC cell lines of AA genotype from the iPSCORE collection, one additional hiPSC cell line of AA genotype (AA-1) was reprogrammed by our laboratory. The cell line was derived from a female lung cancer patient in the age range 30–40. The written informed consent from the donor of the cell line was obtained for this study under Indiana University Institutional Review Board approved protocol #1505859025. Blood cells were expanded using Erythroid Progenitor Reprogramming Kit (StemCell Technologies) and reprogrammed to hiPSCs using Epi5 Episomal iPSC Reprogramming Kit (Thermo Fisher Scientific) per manufacturer’s instructions. The reprogrammed hiPSC line, AA-1, has over 90% expression of all three pluripotency markers Nanog-1, Oct3/4, and Sox2, similar to the eight hiPSC cell lines from the iPSCORE collection. A normal karyotype (46, XX) was also confirmed by G-banded cytogenetic analysis. Cells were routinely maintained in mTESR Plus (StemCell Technologies) on growth factor-reduced Matrigel (Corning) at 9 µg/cm^2^ culture area and passaged every 3–4 days using Dispase (StemCell Technologies). Cell lines were used between passages 20 and 60.

### Generation of the *rs28714259* locus deletion hiPSC lines via CRISPR-Cas9

A pair of CRISPR sgRNAs targeting a 287 bp intergenic region surrounding *rs28714259* were designed at http://crispr.mit.edu/. No SNPs in linkage disequilibrium with *rs28714259 by LDproxy analysis*, or any other known regulatory sequences, were present in this region. sgRNA sequences are: sgRNA-L(upstream): 5′-TGAGTCAAATAAGATGCAGG-3′, and sgRNA-R (downstream): 5′-GCCATATATCTAGGGCCATG-3′. Distance of cut sites to *rs28714259* is 57 bp (upstream) and 230 bp (downstream). Both sgRNAs were predicted to have no off-target sites by the CRISPR RGEN tool Cas-OFFinder (http://www.rgenome.net/cas-offinder/). Each sgRNA was cloned to pSpCas9(BB)−2A-Puro (PX459) V2.0 plasmid (Addgene #62988). The GG-2 hiPSC line which is homozygous of the *rs28714259* major alleles (GG) was used as the parental cell line for genome modification. hiPSCs were detached using Accutase (StemCell Technologies). 2 × 10^6^ cells were co-transfected with 5 μg of PX459 plasmid for each sgRNA using 4D nucleofector (program CB-150) and the P3 Primary Cell Kit (Lonza) per manufacturer’s instructions. The empty pX459 vector was transfected separately as a negative control. hiPSCs were subsequently transferred back to mTESR Plus medium supplemented with 10 μM ROCK inhibitor (Selleck). 24 h post-transfection, cells were subjected to 1 µg/ml puromycin selection for 16 h and allowed to recover for a week. Resistant colonies were picked and expanded for genotyping. Genotyping was performed by PCR using DreamTaq Polymerase (Thermo Fisher Scientific) per manufacturer’s instructions. The following primers were designed 1100 bp upstream and 1187 bp downstream of *rs28714259* to detect successful deletion: 5′-GCAAAATAGATTCTTCAAGTGCTG-3′ (forward) and 5′-GCAGAAAAAGATAAATGAGCAAAGT-3′ (reverse). Un-edited genomic DNA such as those from the vector control clones generated the full length 2287 bp band whereas clones with one or both alleles modified generated the truncated 2 kb band. Clones with both the full length and the truncated bands were selected as the heterozygous deleted (+/−), and clones with only the truncated band were selected as the homozygous deleted (−/−). Deletion in the modified clones was confirmed by deep sequencing (Supplementary Fig. 2). Two clones each of the vector control (+/+), heterozygous deleted (+/−), and homozygous deleted (−/−) genotypes were used in the study.

### Cardiac differentiation of hiPSCs

hiPSC lines intrinsically polymorphic at *rs28714259* or modified by CRISPR-Cas9 were differentiated to cardiomyocytes by small molecules modulating the WNT signaling pathway^39,71^. Most media used was CDM3-based media consisting of RPMI1640 (Corning) supplemented with 500 µg/ml oryza sativa-derived recombinant human albumin (Sigma–Aldrich) and 213 µg/ml L-ascorbic acid 2-phosphate (Sigma–Aldrich). Briefly, hiPSCs at ~75% confluence was switched to CDM3 Media supplemented with 6 µM CHIR99021 (Selleck) (Day 0). 48 h later (Day 2), media was changed to CDM3 supplemented with 2 µM Wnt-C59 (Selleck). Media was changed on Day 4 and every other day to basic CDM3. Contracting cells were noted from Day 7. From Day 10–14, contracting cells were further selected by CDM3-L media made with RPMI1640 without glucose (11879-020, Life Technologies), supplemented with 500 µg/ml recombinant human albumin, 213 µg/ml L-ascorbic acid 2-phosphate, and 4 mM L-lactic acid (Sigma–Aldrich). At day 15–25, surviving contracting cells were matured by switching to CDM3-M media based on CDM3-L, with additional supplement of 10 mM D-galactose (Sigma–Aldrich), 1 mM sodium pyruvate (Life Technologies), 20 µg/ml insulin (Life technologies), 1x chemically defined lipid concentrate (Life Technologies), and 20 ng/ml tri-iodo-L-thyronine (Sigma–Aldrich)^71,72^. No corticosteroid (including dexamethasone) was present in the media used for the differentiation, maturation, or maintenance of iPSC-derived cardiomyocytes. Cells were used for subsequent analysis between Day 26–40.

### Electrophoretic mobility shift assay (EMSA)

hiPSC-CMs or MCF-7 breast cancer cells were treated with 100 nM dexamethasone for 2 h and nuclear protein were extracted. EMSAs were performed with hiPSC-CM nuclear extracts using the LightShift Chemiluminescent EMSA Kit (Thermo Fisher Scientific). The 33 bp oligonucleotide probes centering on *rs28714259* with or without biotin label were synthesized by Integrated DNA Technologies. Sequences are 5′-ttctgatctttgtttt(G/A)cttttcttggcttctg-3′, where uppercase G/A represents *rs28714259*. For supershift assay, EMSA was performed using MCF-7 nuclear extract under the same condition. Glucocorticoid receptor antibody (Abcam ab225886, 1:250 diluted) was incubated with the binding reaction for an additional 30 min for supershift detection. The binding complexes were resolved on a 6% polyacrylamide gel and binding detected by chemiluminescence.

### Chromatin immunoprecipitation (ChIP) assay

ChIP assays were performed in hiPSC-CMs using Magna ChIP Protein A+G Magnetic Beads (Millipore Sigma). Three hiPSC-CM lines intrinsically polymorphic at *rs28714259* (GG-2, GA-1 and AA-1) were pretreated with 100 nM dexamethasone for 2 h. DNA was pulled down by GR antibody (Abcam ab225886, 1:250 diluted). Quantitative RT-PCR was performed on both input and immunoprecipitated DNA with the following primers for a 110 bp fragment centering on *rs28714259*: 5′-ATTTGACTCAGCAGCACTCT-3′ (forward) and 5′-CAGCCCATGAAACACAGTGA-3′ (reverse). Primer sequences for the GILZ promoter region as a positive control were: 5′-GCACTGATTCATGGGTACTGG-3′ (forward) and 5′- ACCAACTCAGGACCAAAGGAG-3′ (reverse). Assays were performed with equivalent amounts of rabbit IgG or rabbit anti-GR antibody. Real-time PCR was performed using Applied Biosystems QuantStudio6 and data collected by QuantStudeio software v1.3. Data were analyzed as the percentage of input DNA and calculated as previously described^73^.

### hiPSC-CMs plating and drug treatment

Matured cardiomyocytes at differentiation day 26 were dissociated using the Cardiomyocyte Dissociation Kit per manufacturer’s instructions and filtered through a 37 µM cell strainer (both from StemCell Technologies). 100,000 live cells were plated into each well of 1:200 matrigel-coated 96-well white-sided clear bottom plates (Nunc) for live imaging and viability assays. 24 h after seeding, cells were pre-treated with vehicle (0.1% ethanol) or 100 nM dexamethasone (Sigma Aldrich) in CDM3 media for 24 h before exposed to vehicle (0.1% DMSO) or 1 µM doxorubicin (MilliporeSigma) in CDM3 media for 24 h. We exposed cardiomyocytes to 1 µM doxorubicin as this was the IC50 of the hiPSC-CMs (Supplementary Fig. 4). This concentration allowed sufficient cell viability to optimally determine the functional impact of *rs28714259*. This study was performed based on the comparison of the following control and treatment groups: control group (Con) was pre-treated with ethanol vehicle before exposure to DMSO vehicle; dexamethasone group (Dex) was pre-treated with dexamethasone before exposure to DMSO vehicle; doxorubicin group (Dox) was pre-treated with ethanol vehicle before exposure to doxorubicin; combination group (DD) was pre-treated with dexamethasone before exposure to doxorubicin.

### hiPSC-CM viability and movie-based contraction assay

CellTiter-Glo Cell Viability assay (Promega) was used to evaluate hiPSC-CM viability after drug treatment. Briefly, 100 μL of reconstituted reagent was added to each well of a 96-well plate and the plate was rocked for 2 min using slow orbital mode then incubated for 10 min at room temperature, all within the Synergy LX reader (BioTek). Six wells with 100 μL of CDM3 media only were used as background controls. Luciferase was read with the Synergy LX reader (BioTek) using 1 s collection time. Viability was normalized to the vehicle-treated cells of each cell line. Statistical analyses were performed using the results of 3 lines/genotype for the intrinsically polymorphic hiPSC-CM lines (GG/GA/AA) and 2 lines/genotype for genome-modified lines [vector control (+/+) /heterozygous deleted (+/−)/homozygous deleted (−/−)], respectively. Experiments with four technical replicates for each hiPSC-CM cell line were conducted. Sample size was *n* = 12 and *n* = 8 for each treatment in the intrinsic and deleted lines, respectively.

For contraction assay, 10x phase contrast images of beating syncytium were captured under 37 °C and 5% CO_2_ using a BioTek Lionheart FX Automated Live Cell Imager. Images were acquired using the fast kinetic mode at 5 ms interval for a total of 15 s in each field using the Gen5 software and converted to movies of 12 fps (frame per second). For contraction analysis, 3 lines/genotype (GG/GA/AA) for the intrinsically polymorphic hiPSC-CM lines and 2 lines/genotype for genome-modified lines [vector control (+/+) /heterozygous deletion (+/−)/homozygous deletion (−/−)] were included. Movies from four technical replicate wells for each cell line/treatment were analyzed and contraction data were extracted from image analysis by the Cellogy Pulse platform (Dana Solutions)^74^. Sample size was *n* = 12 and *n* = 8 for each treatment in the intrinsic and deleted lines, respectively.

### Luciferase assay

An ~2 kb fragment centering on *rs28714259* was cloned to the pGL4.26 luciferase vector containing only a minimal promoter (Promega). Fragments containing the major (G) or risk (A) allele were amplified using phusion polymerase (ThermoFisher) from genomic DNA of intrinsically polymorphic hiPSC cell lines of *rs28714259* GG and AA genotypes, respectively. Primers used were: 5′-ccgctcgagGCAAAATAGATTCTTCAAGTGCTG-3′ (forward) and 5′- cccaagcttACATACCAAATTGAAGGGCTGG-3′ (reverse). Sequences in lower case represent XhoI and HindIII sites included for cloning into luciferase vector. The final luciferase G- and A-constructs were confirmed for sequence accuracy by sanger sequencing.

hiPSC-CMs in 96-well plates (100,000/well) were transfected with empty pGL4.26 vector (EV), major allele (G)-, or risk allele (A)-constructs using Lipofectamine 3000 transfection reagent (Invitrogen). The renilla luciferase reporter plasmid pRL-TK (Promega) was co-transfected as a control for transfection efficiency. 24 h after transfection, cells were treated with 100 nM dexamethasone for 24 h. Luciferase activity was measured using the Dual-Glo Luciferase Reporter Assay (Promega). Relative luciferase activity was first normalized to renilla luciferase and then normalized to that of the empty vector-transfected (EV) group without dexamethasone stimulation. Three independent experiments were performed with three technical repeats for each construct transfection (*n* = 9).

### RNA sequencing gene expression and pathway analysis

Nine hiPSC-CM lines intrinsically polymorphic (3 lines/genotype) at *rs28714259* were treated with vehicle or 100 nM dexamethasone for 24 h followed by vehicle or 1 µM doxorubicin treatment for 24 h as described above. One independent biological repeat was performed for the GG-2 cell line and total sample size was *n* = 10. Total RNA was extracted using the RNeasy Mini Kit (Qiagen), flash frozen in dry ice and stored at −80 °C until analyzed. Quality control, cDNA synthesis, library preparation (total transcriptome), and sequencing were performed by Center for Medical Genomics at Indiana University School of Medicine. Libraries were sequenced on a NovaSeq 6000 (Illumina) and approximately 80–100 million reads per library were generated. All sequenced libraries were mapped to the human genome (hg38) using STAR (v2.5.2b). Quality control of sequencing and mapping results were summarized using fastqc (v0.11.5) and MultiQC (v1.9). Genes with raw count <20 in 20 or more samples were removed^75,76^. Differential expression analysis was performed using the DESeq2 (v1.24) package in R (v3.6.1). Log2 fold change (Log2FC) was used to interpret gene expression changes as a result of each drug treatment and calculated based on the combined data from the nine intrinsically polymorphic hiPSC-CM lines. The variancePartition (v1.14.1) was used to assess the interaction between *rs28714259* genotype and drug treatment using a linear mixed model with cell line as a random effect. A positive interaction Log2FC indicates risk (A) allele was associated with higher gene expression level after treatment compared to major (G) allele, while a negative value indicates risk (A) allele was associated with lower gene expression level. To identify key biological pathways and gene networks enriched in the differentially expressed genes, ingenuity pathway analysis (IPA) was performed and the Log2 fold change (Log2FC) was used to interpret gene expression changes. For the effect of doxorubicin (DOX) or dexamethasone/doxorubicin combination (DD), genes with |Log2FC| > 0.5 and *P*-value < 0.05 were included in IPA analysis. To evaluate the impact of dexamethasone-pretreatment under the dexamethasone/doxorubicin combination setting, the Log2FC(DOX) of each gene was subtracted from Log2FC (DD) to generate ΔLog2FC. A positive ΔLog2FC indicated an increase in gene expression by dexamethasone pretreatment while a negative value indicated a decrease. Only genes with a *P*-value < 0.05 in both the DOX group and the DD group and a|ΔLog2FC| > 0.3 were included in IPA analysis. For IPA analysis of pathways impacted by *rs28714259* genotype in both DOX and DD group, genes with an interaction |Log2FC| > 0.5 and *P*-value < 0.05 were included. Heat map gene expression were based on normalized counts. Data from one independent biological repeat of the GG-2 cell line was randomly selected for inclusion in the heatmaps. Differential gene expression after treatment for each sample was normalized to its baseline (Control) expression. Colors are scaled by row. Data from one biological repeat of the GG-2 cell line was shown in the heatmaps for clarity.

### Statistical analyses

Comparisons were conducted using an unpaired, two-tailed Student’s *t*-test in Microsoft Excel version 2209 or Prism 8 (GraphPad). Significant differences were defined by *P* < 0.05. Data were presented as mean ± standard deviation (SD). A two-way ANOVA followed by Tukey’s post hoc test was used to determine significant changes in hiPSC-CM viability, contractility, and beat rate between doxorubicin-only and dexamethasone/doxorubicin combination among cell lines of different rs28714259 genotypes. All experiments were performed independently at least three times. For RNA sequencing data analysis, a negative binomial generalized linear model (two-sided test) were applied in DESeq2. The p-values were then corrected for multiple hypothesis testing using the False Discovery rate (FDR) approach of Benjamini-Hochberg (B-H). For pathway analysis, a B-H adjusted p-value was calculated in IPA to account for multiple comparisons. The B-H adjusted *P*-value < 0.05 was considered as statistically significant.

### Reporting summary

Further information on research design is available in the Nature Portfolio Reporting Summary linked to this article.

## Supplementary information

Supplementary Information

Description of Additional Supplementary Files

Supplementary Movie 1

Supplementary Movie 2

Supplementary Movie 3

Supplementary Movie 4

Supplementary Movie 5

Supplementary Movie 6

Reporting Summary

## Data Availability

The RNA-seq data generated in this study have been deposited at SRA with the BioProject identifier PRJNA736968. All data which are needed to derive the conclusions in the paper are available in the paper and the Supplementary Information/Source Data file. The rs28714259 genotype data of iPSC lines is part of the whole genome data deposited by the iPSCORE project at dbGaP (accession number: phs000924, under restricted access controlled by NIH. The names of the cell lines used and their rs28714259 genotype may be shared with other researchers with independent, approved Project Request from their institutions for access to phs000924 per NIH regulations. Source data are provided with this paper.

## Source data

Source Data
