# Supplementary material for: A non-coding GWAS variant impacts anthracycline-induced cardiotoxic phenotypes in human iPSC-derived cardiomyocytes
Source: Nat Commun. 2022 Nov 22;13:7171. doi: 10.1038/s41467-022-34917-y (PMC9684507; doi:10.1038/s41467-022-34917-y)
Supplement: Supplementary file 1 — Supplementary Information [file 41467_2022_34917_MOESM1_ESM.pdf]

Supplementary Information

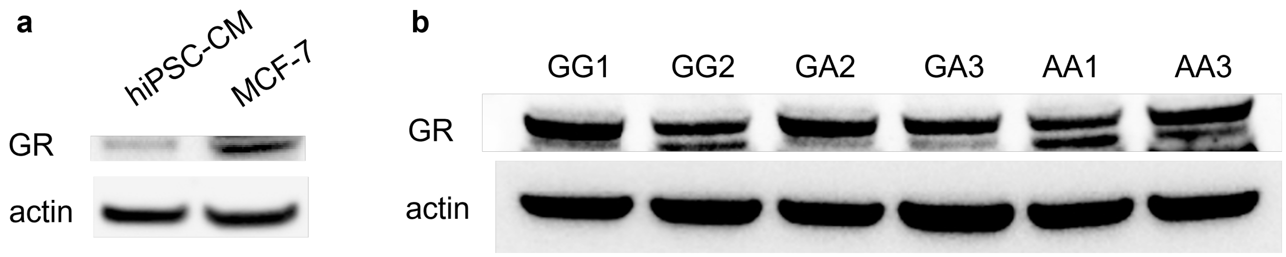

Supplementary Figure 1. Glucocorticoid receptor (GR) expression levels in hiPSC-CMs and MCF-7 breast cancer cells. a-b, GR expression levels were analyzed by western blotting using whole cell lysate from hiPSC-CMs of different *rs28714259* genotypes and MCF-7 breast cancer cells. At least three independent experiments were repeated with similar results. Source data for suppl. fig 3a-b are provided as a Source Data file.

Major (G) AGAAACA TGTTTTG

1 2 3 4 5 6 7   5 6 7 8 9 10 11

Dissimilarity	3.76%	3.76%
RE equality	0.01501	0.01501
RE query	0.06477	0.06477

Minor (A) AGAAACA TGTTTTA

1 2 3 4 5 6 7   5 6 7 8 9 10 11

Dissimilarity	3.76%	N/A
RE equality	0.01501	N/A
RE query	0.06477	N/A

Supplementary Figure 2. PROMO *in silico* prediction of GR binding at *rs28714259* region. Two overlapping GR binding half sites are predicted by PROMO 3.0.2. *rs28714259 (position 11)* and are highlighted in yellow. Dissimilarities to canonical GRE are shown for major and minor allele genotypes.

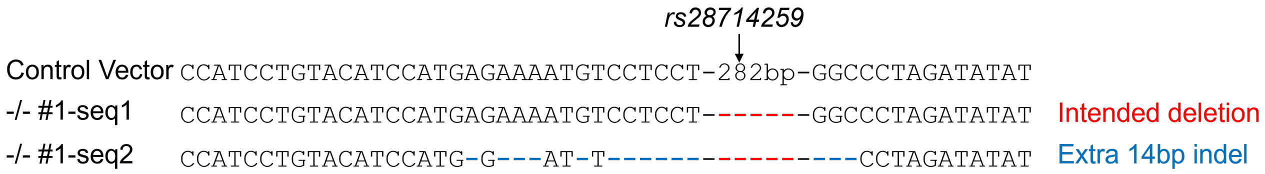

Supplementary Figure 3. Deep sequencing of Crispr-Cas9 mediated genomic deletion of rs28714259 region in hiPSC-CMs. 500bp amplicon surrounding rs28714259 deletion site was sequenced via Amplicon-EZ by Azenta. One homozygous deletion clone has the intended deletion in one copy (seq1) and an extra 14bp indel in the other copy (seq2). The 14bp indel does not contain any known GRE or SNPs in LD with *rs28714259*.

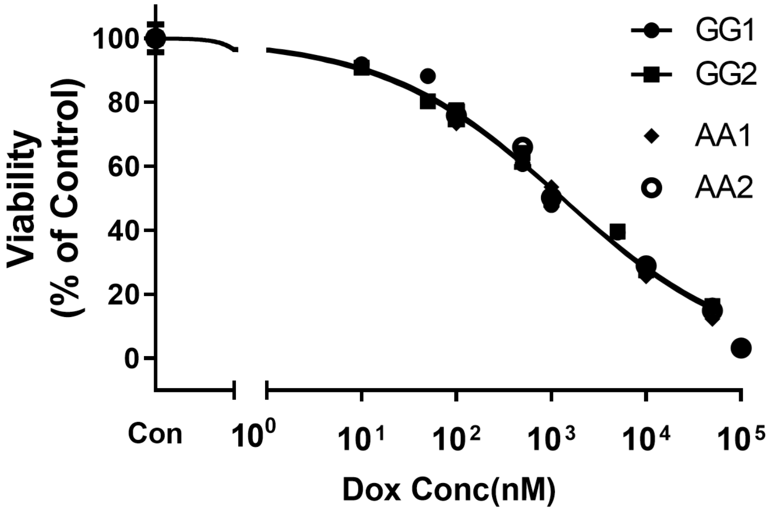

Supplementary Figure 4. Characterization of doxorubicin-induced cell death in hiPSC-CMs. Cell viability of hiPSC-CMs was assessed by measurement of ATP levels using CellTiter-Glo® after 24 h of doxorubicin treatment. Source data are provided as a Source Data file.

Supplementary Table 1 (A-B). Genes and pathways differentially regulated by doxorubicin treatment over vehicle control in cardiomyocytes. (A) Expression fold change of known doxorubicin response genes in cardiomyocytes. Tests for differential expression with an FDR < 0.05 (Benjamini-Hochberg corrected) were considered statistically significant. **(B)** Top pathways differentially regulated by doxorubicin treatment identified by Ingenuity Pathway Analysis. Benjamini-Hochberg (B-H) adjusted p-values were calculated to account for multiple comparisons, and the B-H adjusted p-value < 0.05 was considered as statistically significant. Dox, doxorubicin.

Supplementary Table 1A

Dox Target Genes	log2FC	FDR
TNFRSF10A	4.95	6.52E-49
TNFRSF10B	2.43	6.60E-39
TNFRSF10D	3.91	1.78E-34
TNFRSF10C	3.53	1.71E-19
FAS	3.06	1.21E-16

Supplementary Table 1B

Top Pathways	# Genes	*P*-value	Adjusted *p*-value
p53 Signaling	36	3.98E-07	2.04E-04
Role of BRCA1 in DNA Damage Response	31	6.48E-07	2.04E-04
Kinetochore Metaphase Signaling Pathway	35	2.75E-06	5.76E-04
Molecular Mechanisms of Cancer	96	1.35E-05	1.78E-03
Adipogenesis pathway	41	1.41E-05	1.78E-03

Supplementary Table 2 (A-B). Top genes and pathways differentially regulated between doxorubicin-only (DOX) and dexamethasone/doxorubicin combination (DD) treatment. Tests for differential expression with an FDR < 0.05 (Benjamini-Hochberg corrected) were considered statistically significant. For IPA analysis, Benjamini-Hochberg (B-H) adjusted p-values were calculated to account for multiple comparisons, and the B-H adjusted p-value < 0.05 was considered as statistically significant.

Supplementary Table 2A

Gene	∆Log2FC	*P*-value (Dox)	FDR (Dox)	*P*-value (DD)	FDR (DD)
GJA4	4.17	2.02E-15	7.18E-14	1.62E-08	1.88E-07
DAND5	-3.36	1.06E-05	5.96E-05	2.71E-13	8.17E-12
BEND5	3.15	1.71E-07	1.43E-06	6.56E-04	2.45E-03
MIR145	3.09	2.98E-13	7.45E-12	6.60E-08	6.71E-07
SPC25	-3.04	4.48E-02	9.14E-02	6.85E-06	4.32E-05

Supplementary Table 2B

Top Pathways	# Genes	*P*-value	Adjusted *p*- value
Breast Cancer Regulation by Stathmin1	57	3.93E-05	1.27E-02
CREB Signaling in Neurons	57	5.20E-05	1.27E-02
Kinetochore Metaphase Signaling Pathway	16	1.43E-04	2.33E-02
cAMP-mediated signaling	26	4.66E-04	5.70E-02
G-Protein Coupled Receptor Signaling	29	7.22E-04	7.07E-02

Supplementary Table 3. Genes identified by Ingenuity Pathway Analysis in the GPCR/cAMP pathway differentially regulated between doxorubicin-only and dexamethasone/doxorubicin combination treatment. Genes were ranked by their differences in expression fold change between combination and doxorubicin-only treatment groups (∆log2FC). Role of each gene as "activator" or "repressor" of the GPCR/cAMP pathway was based on the IPA analysis. Tests for differential expression or interaction with an FDR < 0.05 (Benjamini-Hochberg corrected) were considered statistically significant.

Gene	∆log2FC	*p*-value (Dox)	FDR (Dox)	*p*-value (DD)	FDR (DD)	Role	Impact on GPCR/cAMP
CHRM1	2.43	3.15E-05	1.58E-04	1.55E-03	5.19E-03	activator	enhance
TBXA2R	1.35	6.89E-03	1.85E-02	1.42E-02	3.49E-02	activator	enhance
AVPR1B	1.05	3.09E-03	9.16E-03	9.44E-05	4.45E-04	activator	enhance
PDE1A	0.79	9.64E-04	3.30E-03	1.59E-02	3.85E-02	repressor	decrease
HTR2A	0.78	1.62E-02	3.84E-02	2.13E-02	4.94E-02	activator	enhance
PRKCG	0.63	6.94E-08	6.37E-07	7.42E-09	9.21E-08	activator	enhance
GRM6	0.5	3.18E-04	1.24E-03	1.39E-03	4.72E-03	activator	enhance
RGS4	0.45	6.61E-07	4.90E-06	2.37E-06	1.67E-05	repressor	decrease
PLCB2	0.43	5.63E-03	1.55E-02	1.66E-02	3.99E-02	activator	enhance
ADRA1B	0.39	3.21E-04	1.25E-03	3.13E-02	6.83E-02	activator	enhance
ADCY8	0.38	1.13E-07	9.87E-07	2.09E-09	2.90E-08	activator	enhance
PDE7B	0.36	3.12E-04	1.22E-03	2.11E-02	4.90E-02	activator	enhance
MAP2K1	0.32	1.21E-03	4.01E-03	6.12E-04	2.31E-03	activator	enhance
ADRA2B	0.31	3.92E-02	8.17E-02	1.64E-02	3.96E-02	activator	enhance
RAPGEF4	-0.34	2.30E-02	5.18E-02	9.46E-04	3.36E-03	repressor	enhance
PDE9A	-0.35	7.23E-03	1.92E-02	3.43E-05	1.81E-04	repressor	enhance
CNR2	-0.35	1.36E-02	3.31E-02	1.71E-02	4.11E-02	repressor	enhance
APLNR	-0.35	1.56E-03	5.02E-03	4.94E-03	1.41E-02	repressor	enhance
AVPR1A	-0.37	1.26E-13	3.34E-12	5.17E-11	9.78E-10	repressor	enhance
GRM8	-0.37	1.21E-10	1.92E-09	1.96E-07	1.79E-06	repressor	enhance
S1PR1	-0.38	1.09E-12	2.50E-11	1.03E-18	7.99E-17	repressor	enhance
ADORA1	-0.39	1.81E-02	4.23E-02	3.80E-03	1.12E-02	repressor	enhance
PDE3B	-0.41	8.02E-04	2.81E-03	4.59E-02	9.36E-02	repressor	enhance
OPRD1	-0.49	1.46E-15	5.37E-14	1.82E-14	6.67E-13	repressor	enhance
DUSP9	-0.64	9.78E-03	2.48E-02	1.32E-02	3.30E-02	activator	decrease
RAPGEF3	-0.66	1.33E-02	3.23E-02	6.57E-04	2.46E-03	repressor	enhance
HTR6	-0.87	1.80E-02	4.20E-02	1.21E-02	3.05E-02	activator	decrease
SSTR3	-1.29	2.76E-02	6.08E-02	9.45E-03	2.46E-02	repressor	enhance
P2RY14	-1.32	2.01E-03	6.26E-03	3.26E-05	1.74E-04	repressor	enhance

Supplementary Table 4 (A-B). Top pathways differentially regulated by the *rs28714259* risk allele following (A) doxorubicin and (B) dexamethasone/doxorubicin combination treatment identified by IPA. Benjamini-Hochberg (B-H) adjusted p-values were calculated in IPA to account for multiple comparisons, and the B-H adjusted p-value < 0.05 was considered statistically significant.

Supplementary Table 4A

Top Pathways	# Genes	*P*-value	Adjusted *p*-value
Mitochondrial Dysfunction	31	7.17E-17	1.62E-14
Glucose metabolism	6	6.27E-05	5.68E-03
HER-2 Signaling in Breast Cancer	15	3.43E-04	2.22E-02
Sirtuin Signaling Pathway	19	5.15E-04	2.92E-02

Supplementary Table 4B

Top Pathways	# Genes	*P*-value	Adjusted *p*-value
Mitochondrial Dysfunction	33	1.33E-16	3.22E-14
Sirtuin Signaling Pathway	26	3.86E-06	6.24E-04
Huntington's Disease Signaling	21	4.12E-05	5.00E-03
Cardiac Hypertrophy Signaling	32	2.27E-04	2.20E-02
Glioblastoma Multiforme	15	3.47E-04	2.40E-02

Supplementary Table 5. Top genes differentially regulated by the *rs28714259* risk allele following dexamethasone/doxorubicin combination treatment. Tx = Treatment. Tests for interaction with an FDR < 0.05 (Benjamini-Hochberg corrected) were considered statistically significant.

Gene	Tx Log2FC	Interaction Log2FC	FDR
ASAP1	0.93	-0.55	0.03383
SLFNL1-AS1	3.46	-2.05	0.03383
HDAC4	1.46	-0.84	0.03383
HECTD4	1.31	-0.60	0.04880

Supplementary Table 6 (A-B). Genes in the cardiac hypertrophy signaling pathway differentially regulated by the *rs28714259* risk allele following dexamethasone/doxorubicin combination treatment identified by Ingenuity Pathway Analysis. (**A**) Effect of the risk allele on gene expression and expected outcome on hypertrophy signaling. Role of each gene as pro- or anti-hypertrophic was based on the IPA analysis. Tests for interaction with an FDR < 0.05 (Benjamini-Hochberg corrected) were considered statistically significant. **(B)** Identified genes with intersection in canonical pathways regulating cardiomyocyte pathophysiology.

Supplementary Table 6A

Gene	Treatment Log2FC (DD)	Interaction Log2FC	Interaction *P*-value	Interaction FDR	Pro-/Anti-hypertrophic	Variant impact on hypertrophy signaling
HDAC4	1.46	-0.84	8.77E-06	2.60E-02	Pro	Decrease
PLD6	3.88	-1.89	4.30E-04	1.98E-01	Pro	Decrease
IGF1R	0.67	-0.5	4.96E-04	1.98E-01	Pro	Decrease
PDE10A	0.63	-0.57	1.36E-03	3.21E-01	Pro	Decrease
EIF4EBP1	-1.82	0.76	1.77E-03	3.23E-01	Anti	Decrease
DIAPH2	0.2	-0.67	2.53E-03	3.62E-01	Pro	Decrease
PRKAR2B	-1.53	0.63	2.81E-03	3.70E-01	Anti	Decrease
TG	4.9	-1.7	3.12E-03	3.76E-01	Pro	Decrease
ITPR1	1.83	-0.8	3.47E-03	3.84E-01	Pro	Decrease
NFAT5	0.64	-0.5	4.30E-03	4.18E-01	Pro	Decrease
HDAC7	0.89	-0.7	5.40E-03	4.41E-01	Pro	Decrease
WNT10B	3.98	-2.14	8.88E-03	4.88E-01	Pro	Decrease
WNT7B	9.54	-2.48	9.80E-03	5.03E-01	Pro	Decrease
GNG7	-0.8	0.63	1.28E-02	5.30E-01	Anti	Decrease
HSPB1	-0.5	0.84	1.29E-02	5.33E-01	Anti	Decrease
RCAN1	1.64	-1.2	1.45E-02	5.48E-01	Pro	Decrease
ADCY7	0.75	-0.62	1.51E-02	5.48E-01	Pro	Decrease
RPS6KA5	2.66	-0.52	1.69E-02	5.53E-01	Pro	Decrease
WNT2B	1.99	-0.81	1.80E-02	5.53E-01	Pro	Decrease
MAP2K6	-2.99	0.7	1.94E-02	5.53E-01	Anti	Decrease
ATP2A2	-1	0.52	2.63E-02	5.86E-01	Anti	Decrease
GNB3	-0.08	-0.75	3.03E-02	6.16E-01	Pro	Decrease
FZD9	3.68	-0.72	3.22E-02	6.25E-01	Pro	Decrease
PRKCG	5.58	-1.66	3.27E-02	6.31E-01	Pro	Decrease
NFATC1	0.08	-0.56	3.59E-02	6.40E-01	Pro	Decrease
NOTUM	0.64	2.04	3.74E-02	6.46E-01	Pro	Increase
FGFR2	2.12	-0.53	3.86E-02	6.51E-01	Pro	Decrease
RAP1B	0.54	-0.51	4.26E-02	6.63E-01	Pro	Decrease
LIF	2.93	-0.73	4.71E-02	6.76E-01	Pro	Decrease
MAPK3	-1.29	0.57	4.84E-02	6.79E-01	Anti	Decrease
TNFSF13B	2.47	-1.07	4.91E-02	6.82E-01	Pro	Decrease

Supplementary Table 6B

Gene	Intersected pathway	Reference
HDAC4	MAPK/ERK5	Choi et al., Mol Cell 2012
IGF1R	MAPK/ERK1/2/ERK5	Nguyen et al., Reproduction 2007; Benito-Jardón, Cancer Research 2019
PDE10A	GPCR/cAMP	Spiwoks-Becker et al, Neuroendocrinology 2011
EIF4EBP1	MAPK/ERK1/2	Qin et al., Cell Cycle 2016
DIAPH2	GPCR	Lartey et al., Reproduction 2009
PRKAR2B	GPCR/cAMP	Weise et al., Molecular Neurobiology 2019
