# Supplementary material for: A non-coding GWAS variant impacts anthracycline-induced cardiotoxic phenotypes in human iPSC-derived cardiomyocytes
Source: Nat Commun. 2022 Nov 22;13:7171. doi: 10.1038/s41467-022-34917-y (PMC9684507; doi:10.1038/s41467-022-34917-y)
Supplement: Supplementary file 2 — Description of Additional Supplementary Files [file 41467_2022_34917_MOESM2_ESM.pdf]

Supplementary Movie 1: Representative live beating video of iPSC-CMs of rs28714259 G/G genotype in control condition.

Supplementary Movie 2: Representative live beating video of iPSC-CMs of rs28714259 G/G genotype 24h after 1 µM doxorubicin treatment.

Supplementary Movie 3: Representative live beating video of iPSC-CMs of rs28714259 G/G genotype 24h after 1 µM doxorubicin treatment. Cells were pre-treated with 100nM dexamethasone before doxorubicin exposure.

Supplementary Movie 4: Representative live beating video of iPSC-CMs of rs28714259 A/A genotype in control condition.

Supplementary Movie 5: Representative live beating video of iPSC-CMs of rs28714259 A/A genotype 24h after 1 µM doxorubicin treatment.

Supplementary Movie 6: Representative live beating video of iPSC-CMs of rs28714259 A/A genotype 24h after 1 µM doxorubicin treatment. Cells were pre-treated with 100nM dexamethasone before doxorubicin exposure.
