# Supplementary material for: A non-coding GWAS variant impacts anthracycline-induced cardiotoxic phenotypes in human iPSC-derived cardiomyocytes
Source: Nat Commun. 2022 Nov 22;13:7171. doi: 10.1038/s41467-022-34917-y (PMC9684507; doi:10.1038/s41467-022-34917-y)
Supplement: Supplementary file 9 — Reporting Summary [file 41467_2022_34917_MOESM9_ESM.pdf]

nature research

Corresponding author(s): Bryan Paul Schneider

Last updated by author(s): May 18, 2022

Reporting Summary

Nature Research wishes to improve the reproducibility of the work that we publish. This form provides structure for consistency and transparency in reporting. For further information on Nature Research policies, see our Editorial Policies and the Editorial Policy Checklist.

Statistics

For all statistical analyses, confirm that the following items are present in the figure legend, table legend, main text, or Methods section.

n/a	Confirmed	
☐	☒	The exact sample size (*n*) for each experimental group/condition, given as a discrete number and unit of measurement
☐	☒	A statement on whether measurements were taken from distinct samples or whether the same sample was measured repeatedly
☐	☒	The statistical test(s) used AND whether they are one- or two-sided<br>*Only common tests should be described solely by name; describe more complex techniques in the Methods section.*
☒	☐	A description of all covariates tested
☐	☒	A description of any assumptions or corrections, such as tests of normality and adjustment for multiple comparisons
☐	☒	A full description of the statistical parameters including central tendency (e.g. means) or other basic estimates (e.g. regression coefficient) AND variation (e.g. standard deviation) or associated estimates of uncertainty (e.g. confidence intervals)
☐	☒	For null hypothesis testing, the test statistic (e.g. *F*, *t*, *r*) with confidence intervals, effect sizes, degrees of freedom and *P* value noted<br>*Give P values as exact values whenever suitable.*
☒	☐	For Bayesian analysis, information on the choice of priors and Markov chain Monte Carlo settings
☒	☐	For hierarchical and complex designs, identification of the appropriate level for tests and full reporting of outcomes
☒	☐	Estimates of effect sizes (e.g. Cohen's *d*, Pearson's *r*), indicating how they were calculated

Our web collection on statistics for biologists contains articles on many of the points above.

Software and code

Policy information about availability of computer code

Data collection	All data collection was performed using commercially available softwares as outlined below:<br>Live cardiomyocyte videos were taken with BioTek Lionheart FX Automated Live Cell Imager and Gen5 software v3.11.<br>Luciferase activity were measured using BioTek Synergy LX reader and Gen5 software v3.11.<br>Real-time PCR was performed using Applied Biosystems QuantStudio6 and data collected by QuantStudeio software v1.3.<br>Gels and blots were imaged with FujiFilm imager LAS-4000 and ImageReader v.2.1.<br>RNA-seq was performed using Illumina NovaSeq 6000 sequencer.
Data analysis	Software used for data analysis is outlined below:<br>Transcription factor binding sites were analyzed with PROMO 3.0.2.<br>Video-based contractility was analyzed with Cellogy Pulse platform VS1.01.<br>All sequenced libraries were mapped to the human genome (hg38) using STAR (v2.5.2b). Quality control of sequencing and mapping results were summarized using fastqc (v0.11.5) and MultiQC (v1.9).<br>Differential expression analysis was performed using the DESeq2 (v1.24) package in R (v3.6.1). The variancePartition (v1.14.1) was used to assess the interaction between rs28714259 genotype and drug treatment using a linear mixed model with cell line as a random effect.<br>Pathway analysis was performed using Ingenuity Pathway Analysis v62089861.<br>Microsoft Excel version 2209 and GraphPad Prism 8 were used for statistical analyses and plotting.

For manuscripts utilizing custom algorithms or software that are central to the research but not yet described in published literature, software must be made available to editors and reviewers. We strongly encourage code deposition in a community repository (e.g. GitHub). See the Nature Research guidelines for submitting code & software for further information.

Data

Policy information about availability of data

All manuscripts must include a data availability statement. This statement should provide the following information, where applicable:

- Accession codes, unique identifiers, or web links for publicly available datasets
- A list of figures that have associated raw data
- A description of any restrictions on data availability

The RNA-seq data generated in this study have been deposited at SRA with the BioProject identifier PRJNA736968. All data needed to evaluate the conclusions in the paper are present in the paper and the Supplementary Information/Source Data file. The rs28714259 genotype data of iPSC lines from the iPSCORE collection is part of the whole genome data deposited at dbGaP (accession number: phs000924) under restricted access. The names of the cell lines used and their rs28714259 genotype can be shared with other researchers with independent, approved Project Request from their institutions for access to phs000924 per NIH regulations.

Field-specific reporting

Please select the one below that is the best fit for your research. If you are not sure, read the appropriate sections before making your selection.

- [x] Life sciences
- [ ] Behavioural & social sciences
- [ ] Ecological, evolutionary & environmental sciences

For a reference copy of the document with all sections, see nature.com/documents/nr-reporting-summary-flat.pdf

Life sciences study design

All studies must disclose on these points even when the disclosure is negative.

Sample size	Pilot experiments were used to estimate the sample sizes such that appropriate statistical tests would have sufficient power. All experiments except RNA-seq analysis were performed using a minimum of three independent repeats with the exact sample sizes indicated in figure legends or the Methods section. Sample sizes for rs28714259 intrinsically polymorphic hiPSC cell lines were largely based on availability of donors and in commercial cell banks.
Data exclusions	For RNA-seq analysis, genes with raw count < 20 in 20 or more samples were removed. This is an established criteria as most RNA-seq analysis use a raw count threshold of 10 to 50 (Reid et al., eLife 2018, and Hsu et al, Toxicological Sciences 2020). References added in Methods section. For contractility analysis, contraction videos determined by the Cellogy Pulse software as "signal of low quality" were excluded.
Replication	All experiments except RNA-seq analysis were performed using a minimum of three independent repeats and in triplicates across cell lines to confirm that the results were reproducible. All attempts for replication were successful. We did not repeat RNA sequencing due to the cost and study results (Yu L, RNA-Seq Reproducibility Assessment of the Sequencing Quality Control Project. Cancer Inform. 2020 May 20;19:1176935120922498. doi: 10.1177/1176935120922498. PMID: 32489246; PMCID: PMC7241209) on the quality control of RNA seq, which reported acceptable reproducibility across samples replicates. For RNA-seq analysis, we used samples from 3 individual cell lines each for the GA/AA genotype (n=3), and 3 individual cell lines for the GG genotype plus one independent biological repeat from one of the GG cell line (n=4). For the effect of doxorubicin/dexamethasone treatment, data from three genotypes were combined for analysis (n=9). For the effect of rs28714259 genotype, data from same genotype were combined and n=3 independent samples for GA/AA genotype, and n=4 for the GG genotype. The conclusions made in the paper are consistent across independent biological samples.
Randomization	Cells were dissociated into single cell suspension and allocated into treatment groups randomly. For RNA-seq, randomization of treatment/genotype groups was employed when loading libraries into the flow cell.
Blinding	Contractility analysis was done with no blinding as it was performed in a fully automated manner by the Cellogy Pulse software. All exclusion of videos due to low signal quality was done irrespective of the group allocation.

Reporting for specific materials, systems and methods

We require information from authors about some types of materials, experimental systems and methods used in many studies. Here, indicate whether each material, system or method listed is relevant to your study. If you are not sure if a list item applies to your research, read the appropriate section before selecting a response.

Materials & experimental systems

n/a	Involved in the study	
[ ]	[x]	Antibodies
[ ]	[x]	Eukaryotic cell lines
[x]	[ ]	Palaeontology and archaeology
[x]	[ ]	Animals and other organisms
[ ]	[x]	Human research participants
[x]	[ ]	Clinical data
[x]	[ ]	Dual use research of concern

Methods

n/a	Involved in the study	
[x]	[ ]	ChIP-seq
[x]	[ ]	Flow cytometry
[x]	[ ]	MRI-based neuroimaging

Antibodies

Antibodies used	Polyclonal glucocorticoid receptor antibody (Abcam, ab225886) was used for the EMSA (1:250), western blotting (1:1000), and ChIP assay (1:250).
Validation	Primary antibody to glucocorticoid receptor was obtained from Abcam and validated extensively by the vendor. Validation information can be found on the vendor website:<br>anti-glucocorticoid receptor antibody (Abcam, ab225886): Rabbit polyclonal antibody suitable for WB, IP, IHC-P, and ChIP-sequencing. https://www.abcam.com/glucocorticoid-receptor-antibody-ab225886.html?productWallTab=ShowAll

Eukaryotic cell lines

Policy information about cell lines

Cell line source(s)	All commercial hiPSC cell lines were obtained from WiCell institute and renamed GG1-3, GA 1-3, and AA 2-3. Disclosing the original names of the commercial cell lines reveals their rs28714259 genotype which is under restricted access from dbGaP, thus can only be shared with approved Project Request from reader's institutions for access to phs000924 per NIH regulations. This is noted in the manuscript. One hiPSC line was reprogrammed in-house from the peripheral blood of a patient with informed written consent and IRB approval. This cell line is named and referred in the manuscript as AA-1.
Authentication	None of the hiPSC cell lines were authenticated. However, the rs28714259 genotype was confirmed by TaqMan SNP Genotyping Assay (#4351379, Thermo Fisher Scientific).
Mycoplasma contamination	All cell lines used in this study were regularly tested with the MycoAlert Detection kit and were negative for mycoplasma contamination.
Commonly misidentified lines<br>(See ICLAC register)	No cell lines used in this study are on the ICLAC list of commonly misidentified cell lines.

Human research participants

Policy information about studies involving human research participants

Population characteristics	One peripheral blood sample from a 35-40 year old, female lung cancer patient with rs289714259 homozygous risk genotype was collected under a protocol for tissue and blood collection to create an annotated biorepository to support future basic/translational research and testing for genomic changes to help provide personalized treatment. The written informed consent from the donor of the cells line was obtained for this study under Indiana University Institutional Review Board approved protocol #1505859025.
Recruitment	Patient was identified through review for this study. Patient provided informed consent to give blood samples for use in this project.
Ethics oversight	Study was approved by the Indiana University Institutional Review Board #1505859025.

Note that full information on the approval of the study protocol must also be provided in the manuscript.
